# Inclusion of the Guinea Pig Cytomegalovirus Pentameric Complex in a Live Virus Vaccine Aids Efficacy against Congenital Infection but Is Not Essential for Improving Maternal and Neonatal Outcomes

**DOI:** 10.3390/v13122370

**Published:** 2021-11-26

**Authors:** Mark R. Schleiss, Claudia Fernández-Alarcón, Nelmary Hernandez-Alvarado, Jian Ben Wang, Adam P. Geballe, Michael A. McVoy

**Affiliations:** 1 Department of Pediatrics, University of Minnesota Medical School, Minneapolis, MN 55455, USA; ferna128@umn.edu (C.F.-A.); hernande@umn.edu (N.H.-A.); 2 Department of Pediatrics, Virginia Commonwealth University, Richmond, VA 23298, USA; jbwang@vcu.edu (J.B.W.); michael.mcvoy@vcuhealth.org (M.A.M.); 3 Divisions of Human Biology and Clinical Research, Fred Hutchinson Cancer Research Center, Seattle, WA 98109, USA; ageballe@fredhutch.org

**Keywords:** cytomegalovirus (CMV), congenital CMV infection, CMV vaccine, guinea pig cytomegalovirus, CMV immune modulation, CMV pentameric complex

## Abstract

The development of a vaccine against congenital human cytomegalovirus (HCMV) infection is a major priority. The pentameric complex (PC) of virion envelope proteins gH, gL, UL128, UL130, and UL131A is a key vaccine target. To determine the importance of immunity to the homologous PC encoded by guinea pig cytomegalovirus (GPCMV) in preventing congenital CMV, PC-intact and PC-deficient live-attenuated vaccines were generated and directly compared for immunogenicity and efficacy against vertical transmission in a vertical transmission model. A virulent PC-intact GPCMV (PC/intact) was modified by *galK* mutagenesis either to abrogate PC expression (PC/null; containing a frame-shift mutation in *GP129*, homolog of *UL128*) or to delete genes encoding three MHC Class I homologs and a protein kinase R (PKR) evasin while retaining the PC (3DX/Δ145). Attenuated vaccines were compared to sham immunization in a two-dose preconception subcutaneous inoculation regimen in GPCMV seronegative Hartley guinea pigs. Vaccines induced transient, low-grade viremia in 5/12 PC/intact-, 2/12 PC/null-, and 1/11 3DX/Δ145-vaccinated animals. Upon completion of the two-dose vaccine series, ELISA titers for the PC/intact group (geometic mean titer (GMT) 13,669) were not significantly different from PC/null (GMT 8127) but were significantly higher than for the 3DX/Δ145 group (GMT 6185; *p* < 0.01). Dams were challenged with salivary gland-adapted GPCMV in the second trimester. All vaccines conferred protection against maternal viremia. Newborn weights were significantly lower in sham-immunized controls (84.5 ± 2.4 g) compared to PC/intact (96 ± 2.3 g), PC/null (97.6 ± 1.9 g), or 3DX/Δ145 (93 ± 1.7) pups (*p* < 0.01). Pup mortality in sham-immunized controls was 29/40 (73%) and decreased to 1/44 (2.3%), 2/46 (4.3%), or 4/40 (10%) in PC/intact, PC/null, or 3DX/Δ145 groups, respectively (all *p* < 0.001 compared to control). Congenital GPCMV transmission occurred in 5/44 (11%), 16/46 (35%), or 29/38 (76%) of pups in PC/intact, PC/null, or 3DX/Δ145 groups, versus 36/40 (90%) in controls. For infected pups, viral loads were lower in pups born to vaccinated dams compared to controls. Sequence analysis demonstrated that infected pups in the vaccine groups had salivary gland-adapted GPCMV and not vaccine strain-specific sequences, indicating that congenital transmission was due to the challenge virus and not vaccine virus. We conclude that inclusion of the PC in a live, attenuated preconception vaccine improves immunogenicity and reduces vertical transmission, but PC-null vaccines are equal to PC-intact vaccines in reducing maternal viremia and protecting against GPCMV-related pup mortality.

## 1. Introduction

Congenital infection with human cytomegalovirus (HCMV) causes considerable morbidity and occasional mortality in newborn infants that acquire infection in utero [1,2]. A preconception vaccine capable of preventing virus transmission to the fetus is considered a major public health priority [3,4,5]. Although the lack of a clear immunological correlate of protective immunity has hampered the development of an HCMV vaccine [6], it seems clear that antibody responses targeting viral envelope glycoproteins, as well as cellular immune responses (CD4+ and CD8 +) targeting structural and regulatory proteins [7], play important roles in protection against acquisition and/or reactivation of infection [8]. In clinical trials, recombinant HCMV subunit vaccines have focused on the immunodominant glycoprotein B (gB) as well as the major CD8+ T-cell target, ppUL83 or pp65 [8,9,10,11]. More recently, the HCMV pentameric complex (PC), consisting of glycoproteins gH (gpUL75), gL (gpUL115), and proteins UL128, UL130, and UL131A, has also emerged as a compelling target for vaccine development [12,13,14,15,16]. Antibodies to gB and PC could be complementary in protecting against HCMV, based on the different roles these factors play in entry. Entry of HCMV into endothelial and epithelial cells utilizes the PC and is mediated by endocytosis in a pH-dependent fashion. In contrast, entry into fibroblasts is believed to be initiated by the interaction of the trimer, consisting of gH, gL, and glycoprotein O (gO), with a cell surface receptor and is non-endocytic and pH-independent [17]. A model has emerged in which gB mediates the membrane fusion event between the viral and host cell membranes, while the gH/gL/gO trimer and/or the PC provide triggering signals to gB. In endothelial and epithelial cells all three are needed (gB, trimer, and PC), whereas in fibroblasts only gB and trimer are needed [18]. The PC promotes a mode of cell-associated spread that resists antibody neutralization, as opposed to the gH/gL/gO trimer, which is broadly required for the infectivity of cell-free virions [17]. Both PC- and trimer-specific antibodies in sera from HCMV-seropositive individuals appear to play roles in limiting infection [19,20,21], although protection against congenital transmission, conferred by neutralizing antibodies targeting the PC and the trimer, is incomplete [22].

In addition to subunit vaccine strategies, vaccines based on live, attenuated strains of HCMV have been examined in clinical trials. One such vaccine is based on the laboratory-adapted Towne strain of HCMV, which proved to generate suboptimal immunity in a number of clinical trials [23,24,25,26]. Subsequent to these trials, genomic characterization of the Towne strain demonstrated that among the mutations acquired upon serial tissue culture passage, a 2-base pair insertion (TT) leading to a frameshift mutation in UL130 was noted [27,28,29,30]. This mutation abrogated the production of the viral PC [31]. These observations support the hypothesis that at least one explanation for the suboptimal efficacy of the Towne vaccine may be its inability to induce antibodies to the PC, which in turn could impair the ability of vaccine immune responses to block viral entry into epithelial/endothelial cells [32]. Strategies to increase the immunogenicity of a live-attenuated vaccine approach include co-administration of Towne vaccine with IL-12 adjuvant [33], generation of “chimeric” vaccines consisting of portions of the Towne genome with the less attenuated Toledo strain genome [34,35], and the generation of conditionally replication-defective vaccines expressing the PC. Once such PC-positive vaccine, V160, was rendered conditionally replication-defective by fusing the destabilizing domain of FK506-binding protein 12 (ddFKBP) to essential viral proteins IE1/2 and pUL51, targeting them to proteasome degradation and blocking replication. This effect can be reversed by a synthetic molecule, Shield-1, that binds to ddFKBP, allowing the vaccine virus to be generated. This vaccine has entered clinical trials [36,37,38,39]. However, no studies have documented whether the inclusion of the PC in a live-attenuated or disabled single cycle (DISC)-based HCMV vaccine enhances protection against cCMV infection.

In order to examine the role of immune responses to the PC in vaccine-mediated protection against cCMV, small animal models using species-specific CMVs are valuable. The guinea pig CMV (GPCMV) is able to cross the placenta and cause fetal infection, making this a useful system to study vaccines against cCMV [40]. Notably, the GPCMV encodes a homologous PC (consisting of proteins gH, gL, GP129, GP131, and GP133) which plays an important role in viral pathogenesis [41,42,43]. Mutations disrupting GP129 expression impair the dissemination of virus in experimentally infected animals [44], possibly mediated through a requirement of this protein for viral macrophage tropism [45]. The roles of the GPCMV PC in mediating cell entry, and as a vaccine target in the congenital transmission model, are less clear. Auerbach et al. demonstrated that the GP129-133 proteins, with gH/gL, form a PC, but that a *GP129-133* deletion mutant was moderately defective in both endothelial and fibroblast cell entry, indicating that the PC function was not specific to endothelial tropism, but was more broadly involved in cell entry, including fibroblasts [43]. Other studies have suggested a role of the GPCMV PC in epithelial/endothelial tropism analogous to the situation with HCMV [46]. Antibodies targeting the GPCMV PC are capable of neutralizing virus in cell culture, and vectored subunit vaccine studies using an MVA virus vector demonstrated an impact of a PC-based vaccine on GPCMV dissemination in dams, and reduced congenital GPCMV transmission to pups, in a pregnancy/challenge model [47]. In this report, we describe efforts that were undertaken to specifically elucidate the impact of the GPCMV PC on vaccine efficacy, in the context of a live, attenuated vaccine approach, against maternal and cCMV infection in the guinea pig vertical transmission model. We also examined the impact of the inclusion of an intact PC on vaccine efficacy using a vaccine with targeted deletions impacting immune modulation genes including a virally-encoded protein kinase R (PKR) antagonist and virally-encoded MHC-1 homologs, putative natural killer (NK) cell evasins.

## 2. Materials and Methods

### 2.1. Guinea Pigs

GPCMV-seronegative outbred Hartley guinea pigs were purchased from Elm Hill Laboratories (Chelmsford, MA, USA), and housed under conditions approved by the Institutional Animal Use Committee (IACUC) policy at the University of Minnesota, Minneapolis (UMN). 

### 2.2. Virus and Cells

Salivary gland-passaged GPCMV virus stocks were prepared in strain-2 guinea pigs as previously described [48]. GPCMV virus r129-Turbo (designated as vaccine PC/intact) is a bacterial artificial chromosome (BAC)-derived variant of N13R10 [48] in which a 4-bp deletion in *GP129* was repaired [49] and the far-red fluorescent protein (RFP) marker TurboFP635 was inserted adjacent to the *cre*-excisable BAC origin of replication [50]. BAC-derived viruses were reconstituted from BACs (see below), propagated, and titered as described [50], using guinea pig lung fibroblast cells (GPL; ATCC CCL158) grown in F-12 medium supplemented with 10% fetal calf serum (FCS, Fisher Scientific), 10,000 IU/l penicillin, 10 mg/L streptomycin (Gibco-BRL), and 0.75% NaHCO_3_ (Gibco-BRL). Cells were screened for mycoplasma using PCR kit 30-1012K (ATCC).

### 2.3. Generation of Live, Attenuated Vaccines

**Figure 1:**
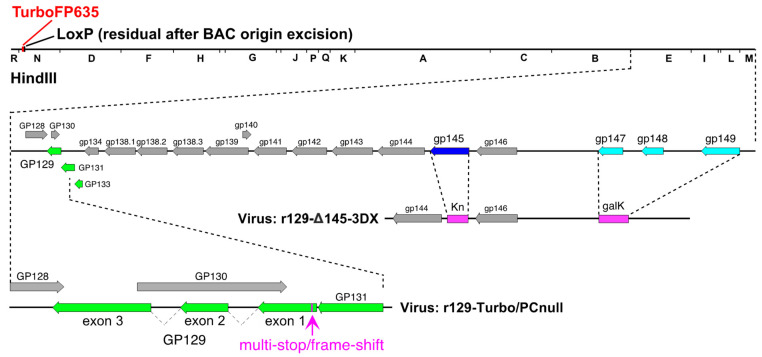
Schematic representation of vaccine candidates evaluated in this study. Genetic features are illustrated for parental PC-intact virus r129-Turbo, PC-null virus r129-Turbo/PC-null, and PC-intact double-deletion virus r129-3DX/Δ145 showing PC subunit genes in green. In r129-Turbo/PC-null the *GP129* open reading frame is disrupted by an 11-bp insertion in exon 1, while in r129-3DX/Δ145 the PC subunit genes are intact but substitutions replace *gp145* (encoding a PKR evasin [50], dark blue) with kanamycin-resistance (*Kn*) and *gp147-149* (encoding MHC I homologs [53], light blue) with *galK*.

Vaccine viruses were constructed in the r129-Turbo background. In virus r129-Turbo/PC-null (designated as vaccine PC/null) an 11 bp multi-stop/frame-shift cassette was inserted near the 5′ end of the *GP129* ORF in order to disrupt *GP129* without disrupting *GP130*, a putative ORF of unknown function on the opposite strand (Figure 1). Two-step galactokinase-mediated recombineering [51] was used as described previously [44]. In step one, a *galK* cassette encoding galactokinase was inserted into *GP129* codon 15 by PCR of plasmid pgalK with oligonucleotide gp129-null-galk-FW (5’-CGCGACGATCTTTAGAACTATATATACAACACGGGTCGTCAAAGATACCGACGACTCACTATAGGGCGAATTGG-3′) and oligonucleotide gp129-null-galk-RV (5’-GATAAAATGCGTGTTATTGTTTTATTGGTTATGTTTTACTATACCCGTCCGCTATGACCATGATTACGCCAAGC-3′), transformation of the PCR product into *E. coli* strain SW102 cells containing BAC N13R10r129-TurboFP635 [52], colony selection on Gal-positive selection plates, and verification of a clone containing the expected *galK* insertion using PCR methods as described previously [44]. 

In step two a 179-bp double-stranded DNA containing an 11-bp multi-stop/frame-shift cassette (TAACTAATTAA) flanked by 84-bp homologies to each side of the *galK* insertion was generated by annealing the oligonucleotides designated gp129-insertion FW (5’-ATCGCTTGCCTGCTAATAAATTGGAACTGGACGTGATAAAATGCGTGTTATTGTTTTATTGGTTATGTTTTACTATACCCGTCCTAACTAATTAACGGTA-3′) and gp129-insertion RV (5’-CTCCACGTATCGTTTGACGTGGTGGAGTGTTGCACGCGACGATCTTTAGAACTATATATACAACACGGGTCGTCAAAGATACCGTTAATTAGTTAGGACG-3′) and extending the single-stranded overhangs by PCR. The product was transformed into SW102 cells containing the *galK* insertion BAC and colonies were isolated using Gal counterselection plates [51]. A clone designated r129-Turbo-129null in which *galK* was replaced by the 11 bp multi-stop/frame-shift cassette was identified by PCR screening and confirmed by targeted Sanger sequencing. 

In virus r129-Turbo/3DX/Δ145 (designated as vaccine 3DX/Δ145) two deletion/substitutions removed *gp147-149* and *gp145* (Figure 1). Deletion of *gp147-149* was achieved by transforming *E. coli* strain SW102 cells containing BAC N13R10r129-TurboFP635 [52] with a PCR product generated by amplifying plasmid pgalK DNA using oligonucleotides 3DX-GALK-FW (5’-GTCGCGATTCAAGTGGCTTTGGAGTAGAGTCGTATCTGAAGCGAATGCCGAACGACTCACTATAGGGCGAATTGG-3′) and 3DX-GALK-RV (5’-CAACGACTTTACGCCGCTGCGCATACTCCAACGCGAGGAACCCAAAAGTTGTGCTATGACCATGATTACGCCAAGC-3′), followed by colony selection on gal-positive selection plates, and verification of a clone in which *gp147-149* sequences were replaced by *galK* sequences using PCR and restriction analysis. Deletion of *gp145* was subsequently achieved by replacing *gp145* with a kanamycin resistance (*Kn*) cassette as described previously [50].

### 2.4. Study Design

Young GPCMV-seronegative female Hartley guinea pigs (*n* = 12/group) were immunized with a two-dose series of candidate vaccines at a one-month interval, using a dose of 1 × 10^5^ PFU delivered subcutaneously (SC). Study groups consisted of: (1) PBS (sham vaccination/negative control); (2) PC/intact vaccine; (3) PC/null vaccine; and (4) 3DX/Δ145 vaccine. On days 7 and 14 following each dose, animals were bled by toenail clips for PCR analysis to examine for vaccine-induced DNAemia. Serum was obtained one week following each dose for ELISA and neutralization studies (described below). Following completion of the immunization series (four weeks after the second vaccine dose) animal mating was commenced, and animals were examined weekly for identifying and dating pregnancy, as described in previous studies [50]. In the late second/early third trimester (ten weeks after the second vaccination, and approximately six weeks following commencement of mating, and at an estimated 45 days of gestational age), the dams were challenged with 1 × 10^5^ PFU of a salivary gland-passaged homogenate of GPCMV (SG-GPCMV) administered by SC route and observed daily until delivery. Two animals failed to become pregnant (one animal each in groups 1 and 4), and serologic responses from these animals were included in the vaccine immunogenicity analyses but not in the final pregnancy outcome analyses. Following delivery, pup tissue was immediately harvested from all dead pups, or within 72 h post-delivery for live-born pups. 

### 2.5. ELISA and Neutralization Assay

Previously described protocols [47] were utilized to generate viral ELISA antigen, with some modifications. GPL cells were inoculated with r129 (PC/intact) GPCMV and antigen was prepared at 7 days post-inoculation by subjecting supernatants to gradient centrifugation as described previously [44] to purify viral particles. Wells were coated with 100 ng/well GPCMV antigen expressed as viral particles, or a negative cellular control antigen, consisting of a lysate from uninfected GPL cells, and then were incubated with serial two-fold dilutions of serum from vaccinated and control animals. Bound guinea pig IgG was detected using peroxidase-conjugated rabbit anti-guinea pig IgG (Accurate Scientific, Westbury, NJ, USA) following the manufacturer’s specifications. ELISA titers were defined as the serum dilution producing an absorbance of >0.1 and twice the absorbance of matching control antigen-coated wells. 

For neutralization assays, RFP-tagged virus r129-Turbo was used to infect GPL cells as described previously [47]. Sera from ELISA-negative naïve animals and a polyclonal anti-GPCMV serum served as negative and positive controls, respectively. That dilution of serum resulting in a >50% reduction in infectious foci in cell culture monolayers was defined as the neutralization titer_50_ (NT50).

### 2.6. Real-Time PCR Analyses

Maternal blood was obtained on days 7, 14, and 21 post-challenge with SG-GPCMV for real-time PCR as described in previous studies [47]. Data were analyzed with the LightCycler Data Analysis Software (version 1.5; Roche, Basel, Switzerland) using standard curves generated by serial dilutions of a GP83 plasmid at known concentrations. Viral load was expressed as genome copies per mL of blood or, for tissue, genome copies per mg of tissue. For blood, the limit of sensitivity for detection in this assay was 200 copies/mL, and for tissue, 4 genome copies/reaction (corresponding to ~2 genomes/mg tissue). For statistical comparison, tissue samples negative in the PCR assay were assigned a value of 1 copy/mg.

### 2.7. Sequence Analysis of Congenitally Transmitted Virus

For tissue samples from congenitally infected pups, sequence analysis was performed to evaluate whether congenital infection was due to SG-GPCMV challenge virus, or caused by the vaccination virus. DNA purified as described in Section 2.6 was amplified by conventional PCR, using primers GP130-FW (5′-GGCGTCGGTATCATTACGGT-3′) and GP130-RV (5′-TCTTTGACGACCCGTGTTGT-3′). Previously, we noted that the original r129 virus that rescued the frame-shift mutation in *GP129* in N13R10 (and which was the parent construct for the vaccines described in this paper) contained mutations near the 3’ end of *GP130* that resulted in R93Q and P97S substitutions in the predicted amino acid sequence of GP130 [44,49]. As these mutations were within the intron between exons 1 and 2 of *GP129* on the negative strand, they did not impact the *GP129* ORF coding sequences, but the presence of these polymorphisms did allow differentiation of vaccine from SG-GPCMV virus. 

PCR was performed using GoTaq^®^ Long PCR Master Mix (Promega, Madison, Wisconsin, USA; catalog number M4021) Reaction Assembly for one reaction, in a 25 μL final volume (10 μM primer concentration; 10 μL template; 12.5 μL GoTaq Mix; H_2_0 to 25 μL). The 522 nucleotide PCR products were evaluated by agarose gel electrophoresis, purified (QIAquick PCR Purification Kit, QIAGEN, Germantown, MA, USA; cat # 28104), and subjected to Sanger sequencing at the UMN Genomics Center. Both strands were sequenced. Sequence analysis was performed with QIAGEN CLC Main Workbench 21.0 and MacVector^®^ Software (version 13.0).

### 2.8. Statistical Analyses

GraphPad Prism (version 8.0) was used for statistical analysis. Pup mortality and transmission were compared using Fisher’s exact test with one-sided comparisons. Pup weights were compared with Dunn’s multiple comparisons. The ELISA geometric mean (GMT) titers for two individual dosing time points and the neutralization GMT titers (following the second vaccine dose) were compared using one-way ANOVA. Parametric data sets included viral load measurements in blood and pup tissue, and these were compared using ANOVA followed by Tukey’s multiple comparison test.

## 3. Results

### 3.1. Generation of Attenuated Vaccines and Evaluation for DNAemia Following Immunization

Previously we described the generation of virus r129 by repair of a 4-bp deletion that disrupts GP129 expression and PC formation in viruses derived from BAC N13R10. Previously published analyses confirmed that these modifications resulted in the expression of the PC [44]. The finding that r129 virions contained GP129 as well as two other PC subunits, GP131 and GP133, while all three were absent from N13R10 virions, indicated that repair of *GP129* restored the formation of the GPCMV PC. Replication of r129 in fibroblasts was unaltered compared to N13R10, but following experimental challenge of immune-suppressed guinea pigs, r129 induced significant weight loss, longer duration of viremia, and dramatically higher (up to 1.5 × 10^6^-fold) viral loads in blood and end organs compared to N13R10 [44]. We further modified r129 to express the RFP marker TurboFP635 to produce r129-Turbo, designated in this study as vaccine virus PC/intact [49]. 

Although previous studies had clearly demonstrated that the GPCMV PC is important for pathogenicity in animals [44], it remained unclear what the importance of this protein complex was with respect to protective immunity against congenital GPCMV transmission, induced by vaccination. Therefore, to determine the importance of the GPCMV PC for inducing protective immunity against cCMV infection, we further modified the PC/intact virus to abrogate PC expression by selectively disrupting the *GP129* ORF with an 11 bp multi-stop/frame-shift insertion (as shown in Figure 1), generating the PC/null vaccine virus. 

Given that r129 is virulent and, like SG-GPCMV, capable of transmission in utero [44], and that r129-Turbo differs by only an RFP cassette and a residual LoxP site following excision of the BAC origin [49], we generated a second virus in which two previously independently characterized deletions were combined to generate a PC-positive but attenuated variant (3DX/Δ145). As reported previously, deletions removing *gp145*, which encodes an antagonist of dsRNA-activated PKR [50,54], or deleting *gp147*, *gp148*, and *gp149*, which encode viral MHC I homologs [53], each profoundly attenuated virulence in the PC-negative N13R10 background. To match these mutations, identical viral sequences were deleted from r129-Turbo to create vaccine virus 3DX/Δ145, in which *gp145* was replaced by a *Kn*-resistance cassette, and *gp147-149* were replaced by *galK* (Figure 1). 

To confirm that these vaccines express or do not express the PC as expected, Western immunoblotting was performed using purified virion particles from PC/null and PC/intact vaccine viruses, using cell lysates from uninfected GPL cells as a negative control for the assays. PC protein expression was detected only in PC/intact virions (data not shown), as previously demonstrated [44]. 

Vaccines PC/intact, PC/null, and 3DX/Δ145 were used as live attenuated vaccines in a pregnancy/challenge study. Groups of 12 female Hartley guinea pigs were confirmed to be GPCMV-seronegative, and were then either sham-immunized with PBS or immunized with two, 1 × 10^5^ PFU SC doses of each vaccine at one-month intervals. To evaluate vaccine-induced DNAemia, blood was obtained 7 and 14 days after the first dose and viral genome copy number was quantified by real-time PCR. Vaccination induced transient, low-grade DNAemia in 5 of 12 animals that received the PC/intact vaccine, 2 of 12 that received the PC/null vaccine, and 1 of 11 animals that received the 3DX/Δ145-vaccine (*p* < 0.05 for PC/intact-immunized, versus the combined attenuated vaccine groups). The single animal in the 3DX/Δ145 vaccine group and the two animals in the PC/null group had DNAemia noted, but only at day 7 post-immunization. Two animals in the PC/intact vaccine group had DNAemia noted only at day 7; one animal in this group had DNAemia noted at both day 7 and day 14; and two animals in this group had DNAemia only at day 14. No animals exhibited any signs of illness (weight loss, ruffled fur) attributable to the vaccine virus following either the first or the second immunization (data not shown).

### 3.2. ELISA and Neutralization Responses

**Figure 2:**
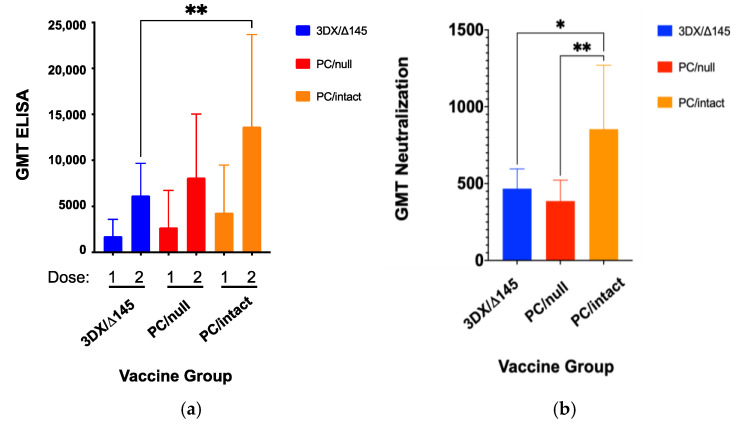
Immune responses to vaccination. Female GPCMV-seronegative Harley guinea pigs (*n* = 12/group) were immunized with a two-dose series of candidate vaccines at a one-month interval, using a dose of 1 × 10^5^ PFU by SC route. Antibody titers (GMT ± 95% confidence intervals [CIs]) by ELISA were compared at day 21 post-immunization for each dose of vaccine (**a**). Mean GMTs were significantly higher for the PC/intact group (GMT 13,669; CI 9640–19,382) when compared to the 3DX/Δ145 group (GMT 6185; CI 4577–8359; *p* < 0.01 vs. PC/intact, ANOVA; Figure 2a), but not significantly different from PC/null (GMT 8127; CI 5498–12,015; P = NS vs. PC/intact). Complement-dependent neutralizing titers, as assessed on GPL cells, were also compared (**b**). Neutralization titers were designated as that dilution of sera that resulted in a reduction of >50% of infectious foci (NT50), using an RFP-tagged, PC-intact virus as described in materials and methods. Data shown consist of GMT ± 95% CIs. Neutralization responses (expressed as GMT ± CIs) were determined using sera obtained at day 21 following the second dose of vaccine and were observed to be higher in PC/intact vaccinees compared to both the PC intact 3DX/Δ145 vaccinees (*p* < 0.05) and PC null (*p* < 0.01) vaccinees. * *p* < 0.05; ** *p* < 0.01.

On day 14 following each immunization dose, GPCMV-specific antibody titers were determined using a previously described ELISA assay [47] modified to use r129 virions, which contain the PC proteins, as antigen. This analysis revealed that mean ELISA titers were significantly higher for the PC/intact group (GMT 13,669; CI 9640–19,382) when compared to the 3DX/Δ145 group (GMT 6185; CI 4577–8359; *p* < 0.01 vs. PC/intact, ANOVA, Figure 2a). Sufficient serum was available from immunized dams to determine the complement-dependent virus-neutralizing titers, after the second dose of vaccine [50]. Consistent with the anti-GPCMV ELISA data, differences in neutralizing titer were also observed (Figure 2b). Dams in the PC/intact-immunized group demonstrated higher mean neutralizing titers compared to either PC/null or 3DX/Δ145 groups (*p* < 0.05; Figure 2b). 

### 3.3. Pregnancy Outcomes after GPCMV Challenge

**Figure 3:**
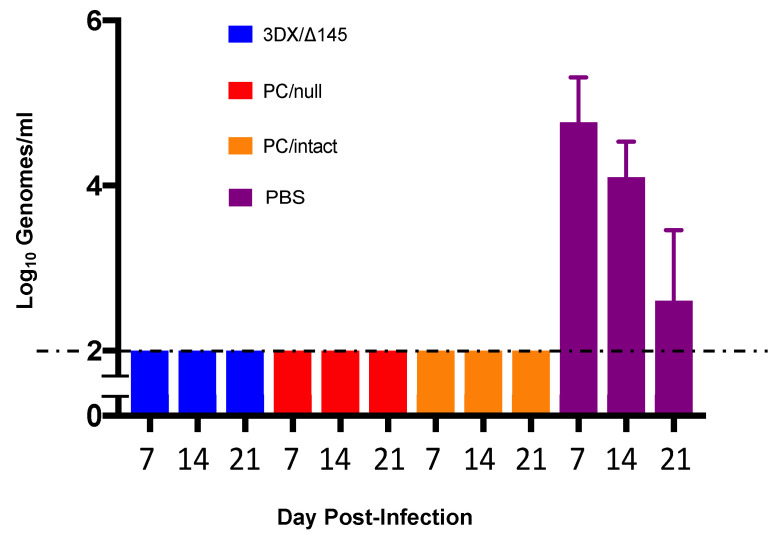
Impact of vaccination on maternal DNAemia. The effectiveness of vaccines, compared to PBS placebo, in reduction in maternal DNAemia following challenge with virulent SG-GPCMV was compared. Whole blood was obtained at 7, 14, 21 days post-challenge and evaluated by real-time PCR. Preconception immunization with all vaccine candidates resulted in complete protection against maternal DNAemia at all time points (limit of detection of assay, 100 genomes/mL). DNAemia (shown as log-transformed mean viral load ± SD) in controls was at its maximum levels at day 7 post-SG-GPCMV challenge (4.8 ± 0.5 log_10_ genomes/mL). All control dams (11/11) had DNAemia on both day 7 and day 14 following SG-GPCMV challenge.

Immunity induced by each vaccine was compared with placebo (PBS) for effectiveness in reducing maternal DNAemia following challenge with virulent SG-GPCMV. Pregnancy was established in all but two animals (one in the control group and one in the 3DX/Δ145 group; Table 1) and pregnant dams were challenged mid-gestation with SG-GPCMV. Whole blood was obtained on days 7, 14, and 21 post-challenge and evaluated by real-time PCR. Remarkably, preconception immunization with all three vaccine candidates resulted in complete protection (below the PCR assay limit of sensitivity) against maternal DNAemia at all time points post-challenge (Figure 3). In contrast, all of the 11 sham-immunized control dams had measurable DNAemia on post-challenge days 7, 14, and 21, with the highest level (peak levels of 1.0 ± 0.3 × 10^5^ genomes/mL) observed on day 7 post-challenge. 

**Figure 4:**
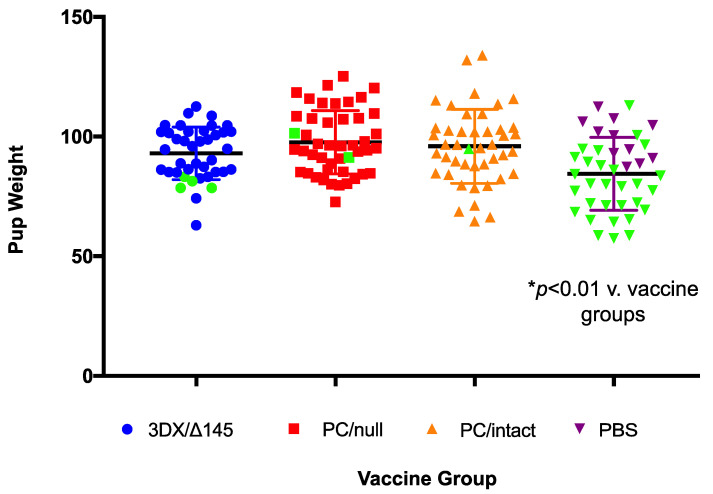
Impact of vaccination on birth weights in pups. The ability of vaccines to improve newborn pup weights, compared to sham-immunized controls, following maternal challenge during pregnancy with SG-GPMV is indicated. For each group, dead pup data points are shown in green marks. Pups born to dams from all vaccine groups had mean weights that were significantly higher than those in the control group (*p* < 0.01).

All vaccines had a positive impact on pregnancy outcome following SG-GPCMV challenge during pregnancy, compared to sham-immunized (PBS) controls (Table 1). Pup mortality in PBS controls was 73% (29/40), and decreased to 2.3% (1/44), 4.3% (2/46), or 10% (4/40) in the PC/intact-, PC/null-, or 3DX/Δ145-vaccinated groups, respectively (*p* < 0.001). There were no significant differences in the duration of pregnancy following the SG-GPCMV challenge in vaccine and control groups, but notably, the newborn pup weights (Figure 4 and Table 1) were significantly lower in pups born to sham-immunized control dams (84.5 ± 2.4 g) compared to the pups born to dams in the PC/intact (96 ± 2.3 g), PC/null (97.6 ± 1.9 g), and 3DX/Δ145 (93 ± 1.7) groups (*p* < 0.01).

### 3.4. Congenital GPCMV Transmission and Pup Viral Load Analyses

**Figure 5:**
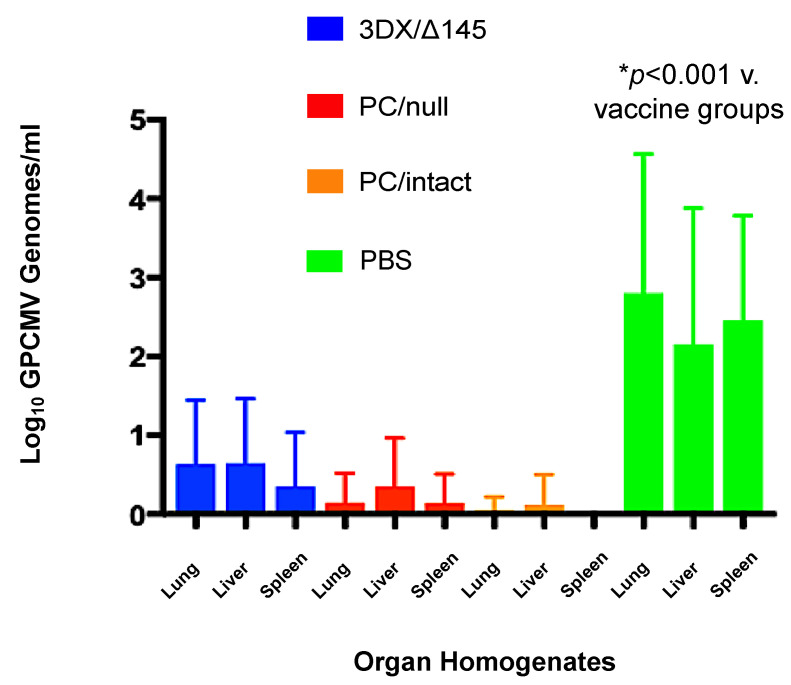
Effect of vaccination on pup viral load. Blue bars, 145/3DX vaccine; red bars, PC/null vaccine; orange bars, PC/intact vaccine; green bars, sham (PBS) vaccine. Viral loads in pup visceral organs (lung, liver, spleen) were determined by qPCR (note only 38 pups were available from the 3DX/Δ145 group). Data shown are log_10_ viral loads (genomes/mg) ± SD. All vaccines conferred reduced viral loads in pup organs compared to PBS (mock) vaccinated controls (*p* < 0.001).

To compare the rates of congenital GPCMV transmission and viral load in infected pups born to dams in the vaccine and control groups, real-time PCR was performed on DNA extracted from liver, lung, and spleen tissues obtained from live-born and stillborn pups. A total of 40 pups were delivered in the 3DX/Δ145 vaccine group, but only 38 were available for PCR analysis. Compared to controls, all three vaccines conferred significantly reduced organ viral loads in pups with confirmed infection (all vaccines, *p* < 0.01; Figure 5). Amongst the vaccine groups, reductions in viral load trended lower in pups born to dams vaccinated with the PC/intact vaccine, but these differences were not statistically significant compared to the PC/null vaccine. As noted in Table 1, considering any positive PCR from any visceral organ as an indicator of vertical transmission for that given pup, congenital GPCMV infections occurred in 11%, 35%, and 76% of pups from dams in the PC/intact, PC/null, and 3DX/Δ145 groups, respectively; in contrast, 90% (36/40) of pups born to control dams were congenitally infected (*p* < 0.0001 versus PC/null and PC/intact vaccine groups; Table 1). The ability of the PC/intact vaccine to protect against vertical GPCMV transmission (39/44) was significantly better than for the PC/null virus vaccine (30/46; *p* = 0.01 vs. PC/null, Fisher exact test). Organ viral loads in infected pups, when compared amongst the vaccine groups, were not statistically different from one vaccine to another, but viral loads, as noted above, were statistically lower in the infected pups from the vaccine groups compared to the pups from the sham-immunized controls (*p* < 0.001; Figure 5). 

### 3.5. Pups Were Congenitally Infected with SG-GPCMV Challenge Virus, and Not the Vaccine Virus

As noted above and in Figure 5, preconception vaccination with all vaccines had a substantial effect both with respect to the reduction in maternal DNAemia and reduction in transmission of GPCMV to pups. However, we wanted to perform additional analyses of the viral DNA recovered in pup tissues to test whether congenital GPCMV transmission was due to the SG-GPCMV used to challenge dams during pregnancy or due to the vaccine virus used to immunize female guinea pigs prior to the establishment of pregnancy.

Toward this goal, we performed sequence analyses on DNA recovered from pup tissues and compared these sequences to those of the vaccine viruses used for immunization. This comparison took advantage of DNA polymorphisms in the 3’ end of *GP130* that resulted in R93Q and P97S substitutions in the predicted amino acid sequence of GP130 [44,49] for vaccine viruses compared to wild-type (SG-GPCMV) sequences. 

**Figure 6:**
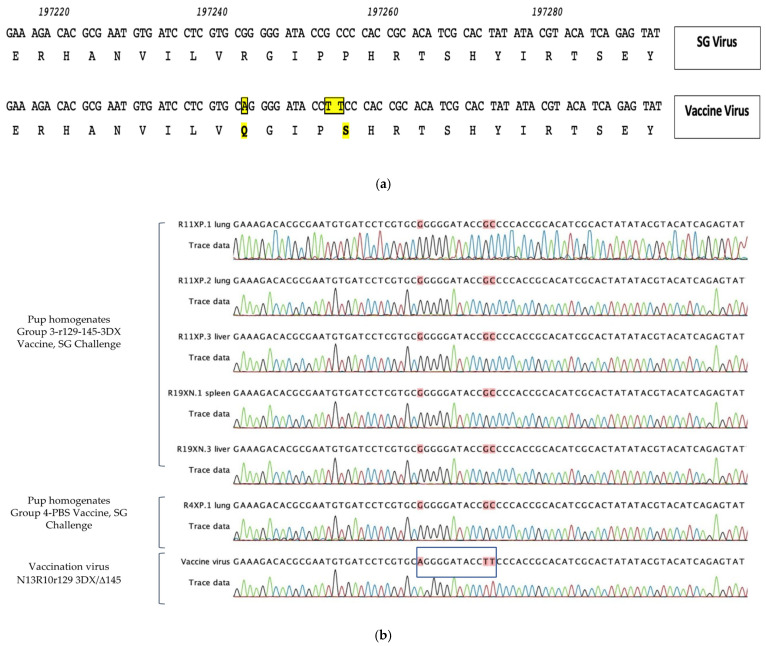
Sequence determination of viral variants identified in organ homogenates of congenitally infected pups in vaccine- and placebo-immunized dams. (**a**) Original r129 virus that rescued the frame-shift mutation in *GP129* in N13R10 was the parent construct for all vaccines used in this study; these viruses contain mutations that result in R93Q and P97S substitutions in the predicted amino acid sequence of GP130 compared to parental SG-GPCMV sequence [yellow shading; 44,49]. Corresponding GenBank coordinates are shown [KC503672]. (**b**) In organ homogenates from pups congenitally infected with GPCMV (both pups born to vaccinated dams [Group 3-r129-145-3DX] and sham [PBS]-immunized dams), congenital transmission occurred with wild-type SG-GPCMV sequence encoding R93 and P97 (red shading) and not with the vaccine variant sequence encoding Q93 and S97, as was confirmed by direct sequencing of vaccine virus (N13R10r129 3DX/Δ145, blue box).

We amplified and sequenced this region for all vaccine viruses in this study (PC/intact vaccine; PC/null vaccine; and 3DX/Δ145 vaccine) and all exhibited both a G->A polymorphism at nucleotide position 197243, resulting in an R->Q aa substitution at codon 93 in *GP130*, and a GC->TT polymorphism at nucleotide positions 197252-197253, which resulted in a P->S substitution in codon 97 (coordinates based on KC503762.1; Figure 6a).

We then amplified and sequenced this region from ten discrete organ samples from pups in the control (unvaccinated, maternal challenge with SG-GPCMV) group. These pups represented ten individual pups from six different litters. All ten amplification products were identical and matched wild-type (SG-GPCMV) sequence, as expected. A representative example from an infected control pup is shown in Figure 6b. Because of the effectiveness of preconception vaccination for all three experimental vaccines (Figure 5), viral loads were very low in many pups (in many cases, just above the threshold of detection). However, we were able to amplify and sequence DNA from five pups in the 3DX/Δ145 vaccine group. In all five pups, the DNA sequence (as demonstrated in the electropherograms, Figure 6b) indicated transmission was due to the SG-GPCMV virus used for maternal challenge, and not from the vaccine virus. 

## 4. Discussion

In this report, we describe a live, attenuated vaccine strategy against congenital CMV infection using a well-established guinea pig model. The primary goal of this work was to test whether the inclusion of the PC in a live virus vaccine resulted in improved protection, but at the same time maintained vaccine safety, compared to a PC-negative vaccine. We had discovered that the parental BAC-derived virus upon which prior vaccine candidates were constructed, N13R10, contained a frame-shift mutation in exon 3 of the *GP129* ORF that abrogated the generation of a functional PC. We, therefore, repaired the mutation, providing, for the first time, a BAC genetic system for constructing mutants in the far more virulent PC-positive r129 background [44]. We further modified r129 to express the far-red fluorescent marker TurboFP635 in virus r129-Turbo. Restoration of the *GP129* ORF resulted in a PC/intact vaccine virus that, although perhaps less virulent than SG-GPCMV, is capable of inducing fetal infection and disease even in the context of naturally GPCMV-seropositive dams [44]. We constructed a PC/null virus by mutating *GP129* (Figure 1) in the PC/intact background to allow a direct comparison of vaccines expressing, or unable to express, the PC for protection against maternal CMV disease and cCMV infection in the GPCMV transmission model.

In previous studies, we demonstrated that vaccination with a live virus vaccine lacking all three MHC I homologs (gp147, gp148, and gp149) reduced pup mortality approximately two-fold [53]. A vaccine lacking the PKR-antagonist gp145 also reduced pup mortality in a dose-dependent fashion, but neither strategy fully protected against congenital GPCMV transmission in the challenge model. To assess whether the attenuation of a PC/intact virus resulting from deletion of genes encoding gp145, gp147, gp148, and gp149 might improve vaccine efficiency, compared to PC/null viruses, we also included the 3DX/Δ145 virus in our vaccine trial. 

All three vaccines induced transient, low-grade viremia that was more prevalent for the PC/intact vaccine than the other two vaccines (5/12 vs. 2/12 and 1/11 combined; *p* < 0.05). When immune responses were compared (Figure 2a), it was observed that ELISA titers were significantly higher for the PC/intact group when compared to the 3DX/Δ145 group (*p* < 0.01 vs. PC/intact), but not significantly different upon comparison to PC/null (P = NS vs. PC/intact). When neutralization responses were compared (Figure 2b), NT50 was observed to be higher in the PC/intact vaccinees when compared to both the PC intact 3DX/Δ145 vaccinees (*p* < 0.05) and PC null (*p* < 0.01) vaccinees. Further investigation will be required to ascertain whether the 3DX/Δ145 vaccine virus produces less PC, or if its attenuation results in less presentation of PC to the immune system due to a reduced magnitude of replication. In spite of these variable differences in immunogenicity, protection against both maternal viremia and pup mortality was substantial in all three vaccine groups (Table 1 and Figure 3). Congenital GPCMV transmission was lowest in the PC/intact group (11%) vs. PC/null (35%) and 3DX/Δ145 groups (76%), respectively, but both the PC/intact and PC/null vaccines were significantly protective (*p* < 0.00001) compared to the 90% transmission rate observed in pups born to the sham-vaccinated controls (Table 1). Among the vaccine groups, viral load reductions trended lower in pups born to dams vaccinated with PC/intact GPCMV, but these differences were not statistically significant compared to the PC/null vaccine (Figure 5). 

Potential limitations to our study include the lack of cytokine and T-cell response data. Since differences in humoral immune response were modest, differences in T-cell responses induced by vaccine candidates may have explained the differences in vertical transmission rates. It would be of interest in future studies to compare neutralizing responses in PC/intact vs. PC/null vaccines using cells of epithelial/endothelial origin versus fibroblasts, but unfortunately, these assays were not performed due to the lack of availability of suitable guinea pig epithelial or endothelial cells at the time these experiments were conducted. However, Auerbach et al. demonstrated that the GPCMV PC plays a role in the entry for both fibroblast and epithelial/endothelial cells [43]; thus, vaccine-induced antibodies to the PC may contribute to the antagonism of viral entry in all cell types, albeit perhaps to a lesser degree than antibodies induced by a gB vaccine which, in direct side-by-side comparison studies, appear to play a more critical role in protection in the GPCMV model [47]. 

Our results suggest that the inclusion of the PC in a live virus vaccine does not greatly augment protection against maternal viremia or pup mortality in the GPCMV model compared to a PC/null viral vaccine. In terms of overall immunogenicity by ELISA, the 3DX/Δ145 deletions had a more detrimental impact on immune response than did the deletion of the PC. However, protection against vertical GPCMV transmission was enhanced in PC/intact compared to PC/null vaccination. Attenuation of PC/intact vaccines with targeted ‘knock-outs’ of viral MHC I homologs (presumed NK cell evasins) and the gp145 PKR evasin reduced vaccine efficacy against vertical transmission, suggesting that the ability to express a functional PC is not sufficient to provide robust protection and, presumably, the ability to replicate in vivo, which was perhaps impaired by the attenuating deletions, also plays an important role for a live vaccine. We observed that vaccination with the PC/intact vaccine induced viremia in 5/12 animals, while only 2/12 PC/null and 1/11 3DX/Δ145 animals had viremia. Moreover, only animals receiving the PC/intact virus demonstrated persistent viremia beyond seven days (3/12, vs. 0/23 animals in the other groups). The improved immunogenicity of the PC/intact virus may have been due to its lack of attenuation, although previous work in this model has shown that viral replication in the maternal compartment (viremia) is not required for immunogenicity and protection against adverse pregnancy outcomes in the context of an attenuated vaccine [53]. Although the inclusion of the PC was not required for reducing maternal viremia or improving pup survival, we did observe reduced congenital transmission in comparing the PC/intact versus PC/null vaccines. The 3DX/Δ145 deletion resulted in a less protective vaccine, even with an intact PC. However, one limitation of our study is that we were not able to evaluate the relative production of PC in wild-type versus 3DX/Δ145 deletion virus. Future studies to confirm that the attenuating mutations do not modify the stoichiometry of PC production are planned. Another potential limitation is that only the 3DX/Δ145 is an attenuated vaccine, in the sense of specific deletions in pathogenesis genes, and there is a positive result noted with this vaccine—it protected against maternal viremia, and against pup death. However, without a direct comparison of 3DX/Δ145 with and without PC, the role of the PC in protection in an attenuated vaccine still requires clarification and is being explored in ongoing studies.

One alternative to attenuated live vaccines is the development of conditional replication-defective whole virus vaccines, which can be engineered to induce immune responses to PC in the absence of viral replication. This approach is being utilized in a clinical trial of the V160 vaccine, a replication-defective strain of HCMV encoding a wild-type PC [36,37,38,39]. Another strategy based on a PC-positive, replication-defective vaccine demonstrated promise in the GPCMV model, although this study did not examine a PC-negative control vaccine in parallel, instead relying on historical controls [55], and hence making it impossible to confirm an essential role for the PC in protection. Our study is the first to report a side-by-side, contemporaneous comparison of PC positive versus PC negative vaccines in the GPCMV model. Future studies of live, attenuated, or replication-defective vaccines for cCMV provide an option for immunization that may confer a greater breadth and depth of immune responses than can be realized by protein, mRNA, or vectored vaccines that induce responses to a limited range of protein targets. Continued examination of proof-of-concept in the GPCMV model with a variety of vaccine platforms should prove useful in informing and directing future trials in this high-priority area of child health research.

## 5. Conclusions

In our study of live, attenuated vaccines in the GPCMV model of congenital infection we made the following observations:

1. The inclusion of the GPCMV PC in a preconception live virus vaccine (PC/intact), compared to a PC/null virus, resulted in improved protection against vertical transmission of the virus (5/44 versus 16/46, *p* = 0.01).
2. PC-intact wild-type virus vaccine was more likely to cause viremia post-immunization (5/12 animals), compared to animals immunized with attenuated PC/null and 3DX/Δ145 viruses (3/33; *p* < 0.05).
3. PC/intact vaccine demonstrated enhanced humoral immunogenicity by ELISA compared to the 3DX/Δ145 vaccine virus.
4. Dams in the PC/intact vaccine group demonstrated higher mean virus-neutralizing antibody titers, assessed on fibroblast cells, compared to either the PC/null or the 3DX/Δ145 groups.
5. All vaccines evaluated in this study protected against maternal DNAemia and pup mortality.
6. When congenital transmission occurred following SG-GPCMV challenge in immunized dams, sequence analysis indicated transmission occurred with wild-type (SG-GPCMV) virus and not vaccine virus.
7. The presence of PC is insufficient, in and of itself, in conferring protection against vertical GPCMV transmission, compared to PC/intact wild-type virus vaccine, in the context of a 3DX [NK evasin]/Δ145 [PKR evasin] double deletion attenuated viral vaccine.
8. Live attenuated and DISC virus CMV vaccination strategies represent a trade-off between the risk of unacceptable reactogenicity and impaired immunogenicity due to over-attenuation. The GPCMV model may prove useful in identifying attenuation strategies that confer protective immune responses, while at the same time improving safety, in future studies.

## Figures and Tables

**Table 1:** Comparisons of pregnancy outcomes in vaccine and control groups.

Vaccine Group	Vaccinated	Pregnant	Total Pups	Live Pups	Pup Mortality	Pup Weight (Grams)	Congenital GPCMV Transmission	Pregnancy Duration Post-SG-GPCMV Challenge
PBS (Control)	12	11	40	11	73%	84.5 ± 2.4	90% (36/40)	24.1 ± 1.2 days
PC/Intact	12	12	44	43	2.3% *	96 ± 2.3	11% (5/44) **	20.8 ± 1.2 days
PC/Null	12	12	46	44	4.3% *	97.6 ± 1.9	35% (16/46) **^,§^	21.3 ± 1.3 days
3DX/Δ145	12	11	40	36	10% *	93 ± 1.7	76% (29/38) ***	20.9 ± 1.3 days

* *p* < 0.001 v. control group ** *p* < 0.00001 v. control group ^§^ *p* = 0.01 v. PC/intact group *** *p* = 0.08 v. control group; only 38 pups were available for PCR analysis.

## Data Availability

Data from this study are stored in a secured site maintained by the UMN Center of Excellence for HIPAA Data (https://it.umn.edu/services-technologies/box-secure-storage; accessed on 24 November 2021). Data are available on request.
